# Differential Response of the Cynomolgus Macaque Gut Microbiota to *Shigella* Infection

**DOI:** 10.1371/journal.pone.0064212

**Published:** 2013-06-05

**Authors:** Anna M. Seekatz, Aruna Panda, David A. Rasko, Franklin R. Toapanta, Emiley A. Eloe-Fadrosh, Abdul Q. Khan, Zhenqiu Liu, Steven T. Shipley, Louis J. DeTolla, Marcelo B. Sztein, Claire M. Fraser

**Affiliations:** 1 Institute for Genome Sciences, University of Maryland School of Medicine, Baltimore, Maryland, United States of America; 2 Department of Pathology, University of Maryland School of Medicine, Baltimore, Maryland, United States of America; 3 Center for Vaccine Development, University of Maryland School of Medicine, Baltimore, Maryland, United States of America; 4 Department of Microbiology and Immunology, University of Maryland School of Medicine, Baltimore, Maryland, United States of America; 5 Department of Pediatrics, University of Maryland School of Medicine, Baltimore, Maryland, United States of America; 6 Department of Medicine, University of Maryland School of Medicine, Baltimore, Maryland, United States of America; 7 Department of Epidemiology and Public Health, University of Maryland School of Medicine, Baltimore, Maryland, United States of America; 8 Department of Microbiology and Immunology, Uniformed Services University of the Health Sciences, Bethesda, Maryland, United States of America; East Carolina University School of Medicine, United States of America

## Abstract

Little is known about the role of gut microbiota in response to live oral vaccines against enteric pathogens. We examined the effect of immunization with an oral live-attenuated *Shigella dysenteriae* 1 vaccine and challenge with wild-type *S. dysenteriae* 1 on the fecal microbiota of cynomolgus macaques using 16 S rRNA analysis of fecal samples. Multi-dimensional cluster analysis identified different bacterial community types within macaques from geographically distinct locations. The fecal microbiota of Mauritian macaques, observed to be genetically distinct, harbored a high-diversity community and responded differently to *Shigella* immunization, as well as challenge compared to the microbiota in non-Mauritian macaques. While both macaque populations exhibited anti-*Shigella* antibody responses, clinical shigellosis was observed only among non-Mauritian macaques. These studies highlight the importance of further investigation into the possible protective role of the microbiota against enteric pathogens and consideration of host genetic backgrounds in conducting vaccine studies.

## Introduction

*Shigella* species represent a group of mucosally invasive bacteria that cause bacillary dysentery, or shigellosis, in humans and nonhuman primates (NHPs) [1]. Only a small inoculum of *Shigella* species is required for disease in humans (∼10–100 bacteria), and the recent appearance of antibiotic-resistant strains and new serotypes is concerning. Worldwide, it is estimated that 90 million cases of food-borne illnesses and over 100,000 deaths are caused by *Shigella* each year [2]. Four different *Shigella* species cause human disease: *Shigella sonnei, Shigella flexneri, Shigella boydii, and Shigella dysenteriae*. While *S. sonnei* accounts for the majority of the cases in the developed world, *S. dysenteriae* 1 is capable of large endemic outbreaks in developing countries, especially within infants and children [1]. Ideally, a successful vaccine against *Shigella* would elicit high immunogenicity against all epidemiologically relevant species without adverse side effects. While both live-attenuated *Shigella* strains and parenteral conjugate vaccine candidates have shown varying degrees of success in human volunteers and NHPs studies, licensed vaccines are not yet available [1].

Vaccine development against *Shigella* has been slowed by discrepancies observed in the efficacy of vaccines evaluated worldwide [1]. For example, the attenuated *S. flexneri* 2a vaccine strain SC602 demonstrated strong immunogenicity and evoked protection in North American volunteers, but it was found to be associated with minimal vaccine shedding and low immune stimulation in volunteers in Bangladesh [3]–[5]. Similar conflicting results between developed and underprivileged global populations have been observed in vaccine studies against polio, cholera, and rotavirus [1], [6], [7]. Development of a successful vaccine against *Shigella* has also been hampered by the lack of a small animal model that mimics human disease [1]. Mice, guinea pigs, and rabbits have been used to assess virulence and screen for vaccine candidates, but these model systems lack the ability to directly predict protection in humans [8]. A more clinically relevant model has been developed with *S. flexneri* in rhesus macaques, *Macaca mulatta*, [9] and *S. dysenteriae* 1 in cynomolgus macaques, *Macaca fascicularis* [10]. However, a large inoculum (∼10^10^ CFU) is necessary to cause bacillary dysentery in non-human primates as compared to humans.

Multiple factors such as diet, nutrition, and host genetics may impact vaccine efficacy [11]. An additional factor that may contribute to the variance in immunogenicity observed between different geographic populations regarding the efficacy of an orally administered vaccine is the composition of the fecal microbiota, which likely represents the bacteria present in the descending colon and rectum. A diverse and rich microbial community within the fecal tract plays an essential function in human health through the acquisition of nutrients, the maintenance of the immune system, and protection from pathogens [12], [13]. Currently, the potential role of the microbiota in vaccine development against enteric pathogens remains unclear.

This study investigates the interaction between the intestinal microbiota and live-attenuated or wild-type strains of *S. dysenteriae* 1 in cynomolgus macaques. The fecal microbiota of wild NHPs has been described in an evolutionary context [14]–[16], as well as in the context to disease in the rhesus macaque model [17]. To date, the intestinal microbiota of the cynomolgus macaque, an animal model frequently utilized in pathogenesis and vaccine research, has not been examined. In this study, we characterized the fecal microbiota in cynomolgus macaques of different geographic origins, and examined the effects of live-attenuated *S. dysenteriae* 1 vaccine strains on the composition of the gut microbiota. Finally, we examined correlations between distinct microbial community profiles and clinical symptoms of infection following challenge with wild-type *S. dysenteriae* 1. These studies represent a detailed examination of microbiota and vaccine interactions in an NHP model.

## Materials and Methods

Additional descriptions of study design parameters, genetic profiling, sample handling, anti-*Shigella* antibody determination, PCR primers and cycling conditions, and data analysis are described in Text S1 containing Supporting Methods.

### Ethics Statement

The study took place in the animal facility of the Program of Comparative Medicine (University of Maryland School of Medicine), an AAALAC-accredited facility. All animals in this study fully participated in the UMSOM “Plan to promote the psychological well-being of nonhuman primates at the University of Maryland School of Medicine”, which includes aspects such as social housing when appropriate, enrichment of physical environment, tactile stimulation, and positive interactions with both conspecifics and humans. This enrichment plan and the IACUC protocol describing this study were specifically approved by the UMSOM IACUC prior to study conduct. Any animal that developed clinical signs referable to dysentery was immediately treated with both fluid support and antibiotics to alleviate discomfort or distress. No animals were euthanized to collect the data presented in this study during the course of the study described.

### Animal Screening and Handling

Male and female cynomolgus macaques (*Macaca fascicularis;* age, 2 to 5 y, n = 33) were purchased from approved vendors as described for each study: study 1 (n = 12) and study 3 macaques (n = 3) from Sierra Biomedical Research (Reno, NV), study 2 macaques (n = 12) from Charles River Laboratories (Houston, TX), and study 4 macaques (n = 6) from Harlan Laboratories, Inc (Indianapolis, IN). Genetic profiling for geographic origin for macaques in studies 1–3 was conducted by Molecular Anthropology Laboratory (Davis, CA). Upon purchase from described vendors, animals screened negative for *Macacine herpesvirus* 1, SIV, simian retrovirus, and simian T-lymphotrophic virus. Animals were quarantined for 3 months before the study began, and tested for intestinal parasites, *Campylobacter, Salmonella, Shigella,* and *Yersinia* spp. prior to study. Only macaques that tested negative for IgG and IgA antibodies to *S. dysenteriae* 1 LPS or titers <1∶50 were used. Macaques were housed in a Biosafety Level 2 containment facility in individual stainless steel primate caging for the study duration, and safe handling practices were conducted while working with *S. dysenteriae* 1. Animals received a commercial primate diet (Teklad 2050, Harlan Laboratories, Indianapolis, IN), with a daily supplement of fresh fruits and vegetables, and given supplemental food enrichment (fruit and nut mix, popcorn, peanuts, granola bars) once or twice weekly. Municipal drinking water was regularly tested and free of any bacterial growth and was provided through an automatic watering system, and was available ab libidum to all macaques throughout the studies. All housing and handling procedures conformed to the *Guide for the Care and Use of Laboratory Animals* and the Animal Welfare Act, and complied to the recommendations in the *Biosafety in Microbiological and Biomedical Laboratories Guide.*

### Study Design

**Figure 1:**
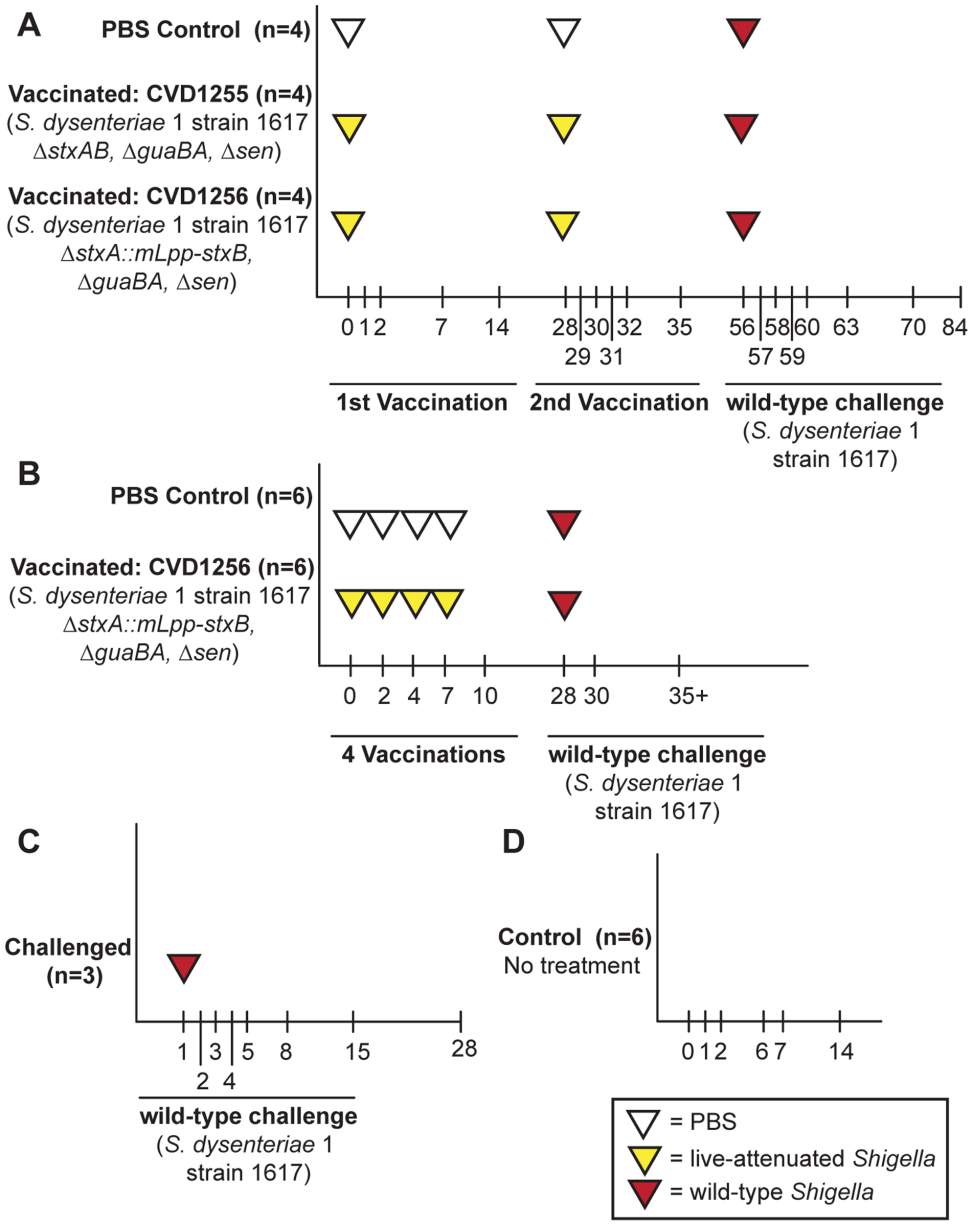
Experimental design for cynomolgus macaque studies. (**A**) Study 1. Macaques were immunized with live-attenuated *S. dysenteriae* 1 strain (CVD1256 or CVD1255) or mock-immunized with PBS on days 0 and 28, followed by wild-type *S. dysenteriae* 1 strain 1617 challenge on day 56. (**B**) Study 2. Macaques were immunized with CVD1256 or mock-immunized with PBS on days 0, 2, 4, and 7, followed by wild-type challenge with *S. dysenteriae* 1 strain 1617 on day 28. (**C**) Study 3. Macaques were challenged with wild-type *S. dysenteriae* 1 strain 1617. (**D**) Study 4. Macaques received no intervention. For all studies, samples were collected at labeled time points (days) for 16 S rRNA pyrosequencing. Immunization types occurred on indicated days (inset box).

Figure 1 illustrates each study timeline (studies 1–4). Macaques from studies 1, 2 and 3 were involved in live-attenuated and/or wild-type *S. dysenteriae* 1 immunization studies to assess the efficacy of live attenuated *S. dysenteriae* 1 vaccine candidates [10], [18]. Two *S. dysenteriae* 1 strain 1617 vaccine candidates were used in immunization studies 1 and 2: CVD1255 (strain 1617 Δ*guaBA* Δ*sen* Δ*stxAB*) and CVD1256 (strain 1617 Δ*guaBA,* Δ*sen* Δ*stxA::mLpp-stxB*). Macaques in studies 1 and 2 were orally inoculated with either phosphate buffered saline (PBS) or 10^11^ colony forming units (CFU) of one of the vaccine candidates, CVD1255 or CVD1256, either two inoculations separated by 28 days (study 1: day 0 and 28), or four times within one week (study 2: days 0, 2, 4, and 7). Both groups were subsequently challenged with *S. dysenteriae* 1 wild-type bacteria strain 1617 (10^11^ CFU via the orogastric route) and monitored on a daily basis for development of clinical signs of disease (study 1 challenged on day 56; study 2 challenged on day 28). Plasma IgA and IgG antibody titers to *S. dysenteriae* 1 LPS were measured by end-point dilution ELISA on days 0, 7, 14, 28, 35, 56, 63, 70, 84 and 98 (study 1) or days 0, 2, 4, 7, 10, 15, 28, 35, and final collection day (36–48) (study 2). Studies 1, 2, and 3 macaques were monitored daily for clinical symptoms of shigellosis ranging from a score of 1 (soft stool) to 4 (severe, bloody diarrhea) as previously described [10]. Study 3 macaques received only wild-type *S. dysenteriae* 1 bacteria with the same protocol. Study 4 received no treatment throughout the course of sample collection. Study design parameters are described in more detail in Text S1.

### DNA Extraction and Pyrosequencing

Sequence data for this project is available under the sequence read archive (SRA) accession number SRA057090, study SRP014769. For all samples, the following pretreatment steps were used to extract total DNA. Samples were resuspended in 1 M KPO_4_, followed by 10 µl Proteinase K and 50 µl 10% SDS. 150 µl Phenol (pH 7.5) was added in conjunction with aggressive bead beating using Lysing Matrix tubes and FastPrep FP120 instrument (QBiogene). For fecal samples collected on days 0–14 in study 1 and all study 4 samples, repeated PCl extractions and ethanol washes were used to filter total DNA. For the remainder of the samples in study 1 and all samples in studies 2 and 3, the ZR Fecal DNA isolation kit (ZYMO Research Corp.) was used to filter total DNA. 50 ng of DNA was used to PCR amplify the V1–V2 region of the 16 S rDNA gene using the universal primers 27F and 338R, with unique barcodes for sample identification [19]. Samples were sequenced using 454 GS FLX or FLX Titanium pyrosequencing chemistry via Life Sciences primer A at the Genomics Resource Center at the Institute for Genome Sciences, University of Maryland School of Medicine according to manufacturer recommendations.

### Data Processing

Mothur v1.19.10 was used to process the generated 16 S rDNA gene amplicons [20]. Sequences were denoised, trimmed and quality-checked before clustering into operational taxonomic units (OTUs) at 97% pairwise identity, and aligned to the Silva reference alignment [21]. The greengenes database (downloaded March 2011) was used to assign taxonomic classification to filtered sequences [22]. The Jensen-Shannon divergence was calculated using an in-house R script, and partitioning around medoids (pam) clustering algorithm in the R package cluster was used to identify community types, or enterotypes [23]. The R packages cluster, vegan, and labdsv were used to conduct principal coordinates analysis (PCoA) and visualize results. In-house scripts, R, and mysql were used to parse data and perform analyses, described in more detail in Text S1. Local similarity analysis (LSA) was used to calculate significant associations among genera and immunological measurements [24]. Cytoscape v.2.8.3 [25] was used to view network analyses.

### Genotype Analysis of MHC Microsatellites

Genomic DNA was extracted from peripheral blood mononuclear cells of all cynomolgus macaques (n = 33) using the Qiagen Dneasy blood and Tissue Kit (catalogue #QGN-69504). We amplified seven previously established MHC-spanning microsatellites, *D6S1691, D6S2741, D6S291, DRACA, DQcar, MICA, MOGc*, and *MICA*, using previously described methods [26]. 10 ng of starting DNA (2 µl of 5 ng/µl DNA) was amplified the Qiagen Multiplex PCR Reaction Kit (cat. # 206143) using 36 cycles of cycling parameters described previously [26], [27]. The PCR product was diluted 50 times following PCR reaction. Genotypes were determined at the Biopolymer Core Facility at the University of Maryland School of Medicine using the Genscan Internal Lane Standard (cat #G9530, ILS-600 Promega-StemElite ID System). A distance matrix of the microsatellite data based on the Lynch distance was calculated using the R package polysat [28], and phylogenetic trees were constructed using Phylip v3.69 [29]. Correlation between microsatellite loci and community type were calculated using an in-house ANOVA R script. For each macaque, community type relative abundance (calculated as the percent time spent in each community type) was normalized using arcsin(sqrt()) transformation, and an ANOVA was used to calculate statistical significance between each community type and the variation present within each locus. Macaques homozygous for an allele were calculated as one, and alleles occurring less than three times within the macaque populations tested were removed from analysis.

## Results

### Characterization of the Cynomolgus Macaque Intestinal Microbiota

To evaluate the intestinal microbiota in cynomolgus macaques, we collected fecal samples from macaques (n = 33) participating in immunization studies at the Center for Vaccine Development (CVD) at the University of Maryland School of Medicine over the course of different treatment and sampling timelines (studies 1–4, Figure 1). Studies 1–3 examined the impact of different immunization protocols with live-attenuated *S. dysenteriae* 1 vaccines and/or challenge with wild-type *S. dysenteriae* 1. In control study 4, macaques received no treatment throughout the course of sample collection, but provide data on the consistency of microbiota parameters over time. A total of 374 fecal samples were collected and the composition of the macaque intestinal microbiota was characterized using 16 S rRNA pyrosequencing of the V1–V2 region (Materials and Methods). The Roche 454 pyrosequencing platform was used to generate a total of 2,779,766 high quality sequences, with an average sequence length of 324 bp and an average of 7,433 reads/sample (Dataset S1).

**Figure 2:**
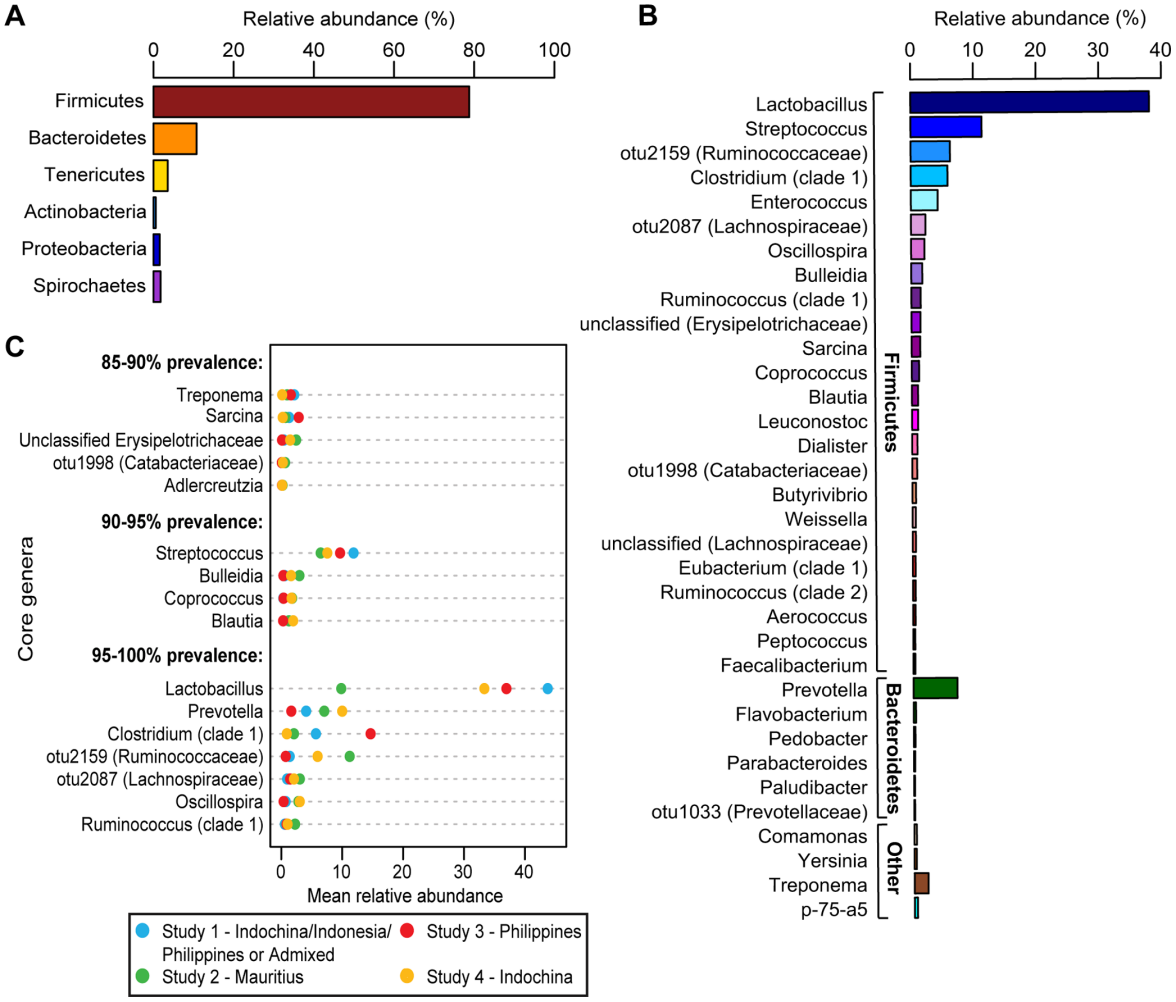
Core fecal microbiota profiles in cynomolgus macaques. Rank abundance plots of (**A**) phyla and (**B**) top 34 most abundant genera. Relative read abundance (within total reads per study) is shown on the y-axis, and genera are listed on the x-axis. (**C**) Core genera (genus-level bacterial groups identified at 85–90%, 90–95%, and 95–100% prevalence rates within the complete sample set of n = 374) are shown on the y-axis. The relative mean abundance for each of these genera within each study group (studies 1–4) is shown on the x-axis, color-coded as indicated in the boxed inset.

To describe the composition of the bacterial communities of the intestinal microbiota in cynomolgus macaques, the Greengenes 16 S rRNA database was utilized to identify 247 different genera [22] (Dataset S1). The two most abundant phyla identified in the cynomolgus macaque fecal microbiota were Bacteroidetes (10.63% of classified sequence reads) and Firmicutes (78.1%) (Figure 2A). While these are the same dominant phyla as those found in the human intestinal microbiota, the cynomolgus macaque microbiota differs from that in humans by its almost 8-fold greater abundance of Firmicutes, as compared to Bacteroidetes [12]. Major genera within the Firmicutes phylum included *Lactobacillus* (38.8%), *Streptococcus* (11.7%), *Clostridium* (clade 1, 6.6%), *Enterococcus* (4.7%), and a *Ruminococcaceae* family member designated as otu2159 (5.7%) (Figure 2B). Typically, one or two of the aforementioned genera represented the dominant genera in these samples at any given time, with other organisms each comprising ≤ 2% of the classified sequences.

We defined a core bacterial community structure within the cynomolgus macaque intestinal microbiota as the genera present in at least 85% of all samples at abundances greater than 0.1% of the total community (Figure 2C). *Lactobacillus, Streptococcus, Prevotella, Clostridium*, and the Ruminococcaceae clade otu2159 represented the most abundant phylotypes across all macaque samples examined, and are considered to be members of the core, being present in ≥90% of all samples collected. While these core genera were present within samples from all four studies, the mean relative abundance of each varied between studies (Figure 2C, Figure S1). The core microbiota also contained several low abundance genera including *Oscillospira* (1.4%) and otu1998 (family *Catabacteriaceae*) (0.55%).

### Community Types of the Cynomolgus Macaque Intestinal Microbiota

**Figure 3:**
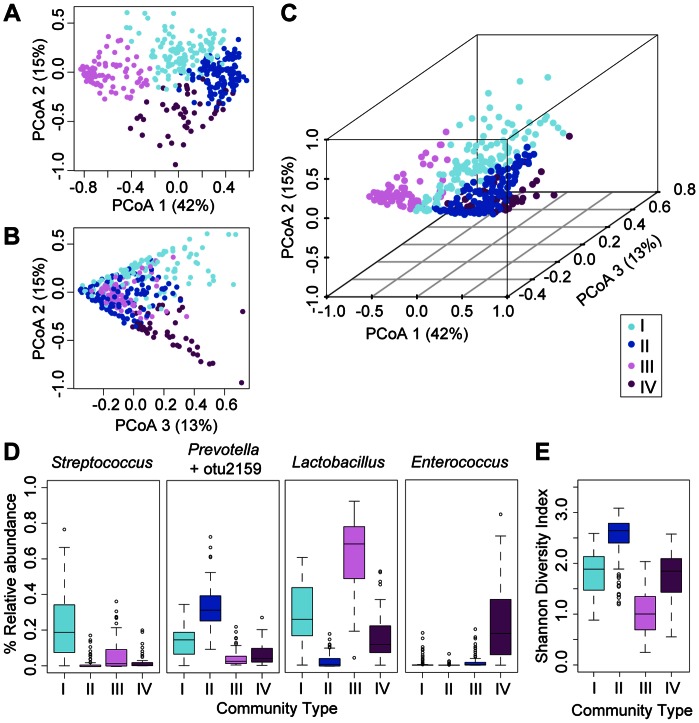
Community types within the fecal microbiota of cynomolgus macaques. (**A–C**) Principal coordinates analysis (PCoA) of axes 1 vs. 2 (**A**), 2 vs. 3 (**B**), and a three-dimensional plot of axes 1–3 (**C**) based on Jensen-Shannon divergence of all samples (n = 374), color-coded by community type clusters identified by the partitioning around medoids (pam) clustering algorithm. Percent variance is shown for each axis. (**D**) Boxplots of the median relative abundance (y-axis) of dominant genera and (**E**) Shannon diversity index (y-axis), observed in each community type (x-axis). Error bars indicate the interquartile range between the first and third quartiles.

**Figure 4:**
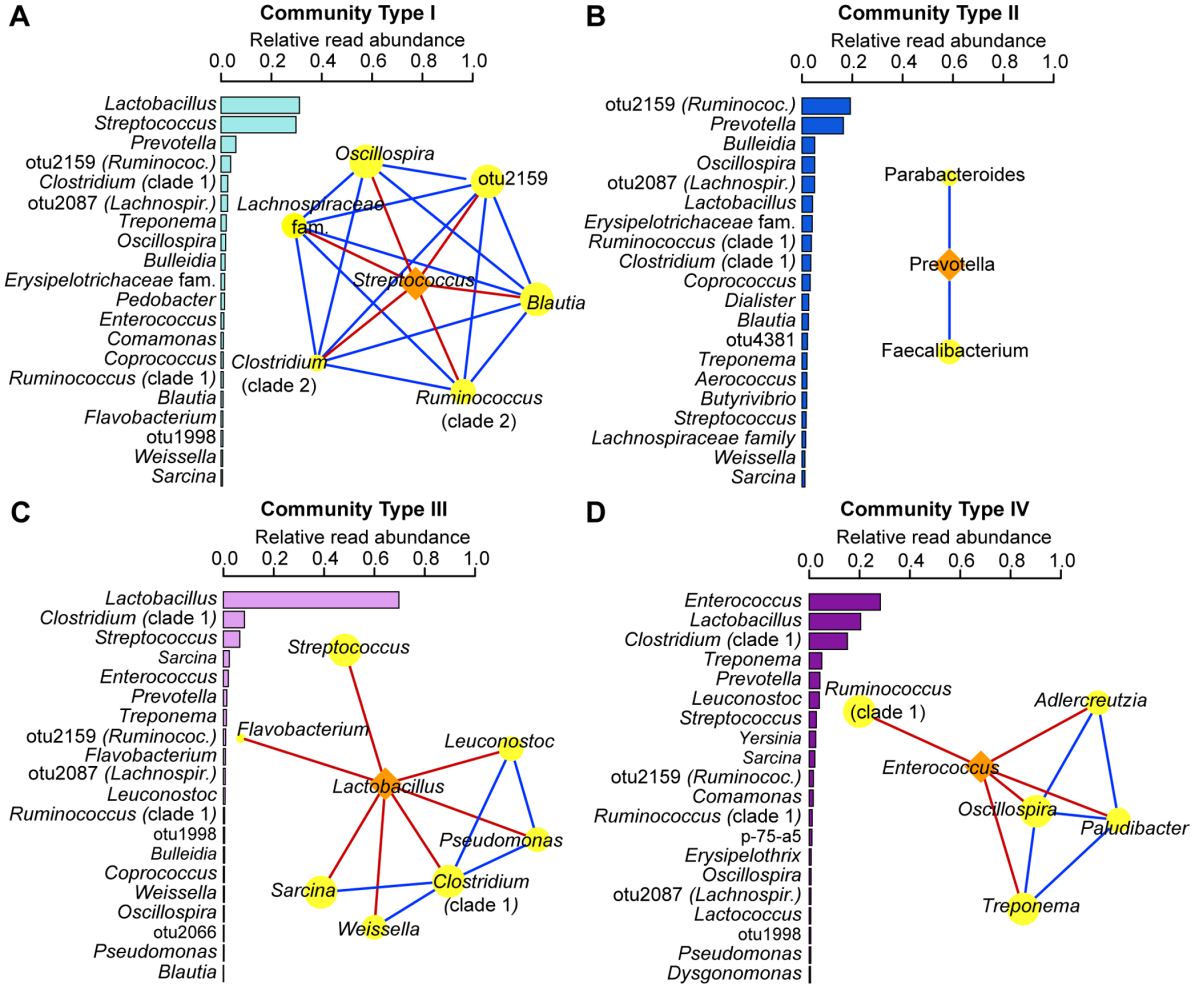
Composition of community types in the cynomolgus macaque fecal microbiota. (**A** to **D**) Rank abundance plots (left) and correlation networks (right) for community types I–IV. For rank abundance plots, the most abundant genera are shown on the y-axis and its relative read abundance is shown on the x-axis, calculated within each community group. Correlation networks were calculated using a Spearman rank correlation coefficient (P<0.001) for the most abundant (pivotal) genera within each community type. Sub-networks (modules) for directly connective genera were identified for each pivotal genera. For each module, the orange diamond vertex indicates the main contributor and yellow ellipse vertices co-occurring genera. Blue and red lines indicate positive and negative correlations, respectively.

To further characterize the cynomolgus fecal microbiota, we investigated whether the microbial communities clustered into specific enterotypes (distinct community types within the gastrointestinal tract), as has previously been described in human fecal samples [23], [30]. We used multidimensional cluster analysis and principal coordinates analysis (PCoA) to compare the calculated Jensen-Shannon divergence for each pairwise comparison of samples in this study. Using the partitioning around medoids (pam) method [23], we identified four distinct clusters, or ‘community types’, analogous to the human enterotypes, designated as community types I–IV (Figure 3A). The average silhouette width suggests a gradient effect for our data set (*S(i)*  = 0.32; 4 clusters), rather than distinct clusters observed in human studies [23]. The majority of samples (87.9%) clustered within community types I–III, and these three community types were found in macaques in all studies, with the exception that the high-diversity community type II was not observed in study 3 (Table 1). Firmicutes members dominated each of these community types, with the exception of community type II, which was characterized by a similar abundance of both a Firmicutes (otu2159, 19.2%) and Bacteroidetes member (*Prevotella,* 16.4%) (Figure 3B, 4B), and a lower abundance of *Lactobacillus* (4.1%) compared to other community types. In contrast, *Lactobacillus* and *Streptococcus* accounted for approximately equal numbers of reads in community type I (31.8% and 29.8%), and *Lactobacillus* accounted for the majority of the reads in community type III (69.8%). Samples clustering into the less abundant community type IV were dominated by *Enterococcus* (28.2%), a prevalent but less abundant member of the core microbiota. These four community types also differed in diversity as measured by the Shannon diversity index (Figure 3C), which is influenced by both richness of genera (number of organisms present) and their relative proportions (evenness). In particular, community type III, which was dominated by a very high percentage of *Lactobacillus* (>60%), exhibited the lowest Shannon diversity, and community type II, which contained several genera in approximately equal proportions, displayed the highest diversity (Figure 3C, 4C).

**Table 1:** Distribution of community types observed in each study.

Community Type:	I	II	III	IV	Total # of samples:
**# samples in study 1:**	79 (36%)	5 (2%)	98 (45%)	38 (17%)	220
**# samples in study 2:**	21 (22%)	66 (70%)	7 (7%)	0	94
**# samples in study 3:**	3 (12%)	0	14 (58%)	7 (29%)	24
**# samples in study 4:**	16 (44%)	13 (36%)	7 (19%)	0	36
**Total # of samples:**	119	84	126	45	374

Number and percentage of samples within each study belonging to community type I–IV. Total numbers within each community type are indicated on the bottom row, and total number of samples on the right end row.

Interactions among genera in a given community type may be essential determinants of functional capabilities and may also influence the stability of a community in response to environmental perturbations. To identify specific relationships between genera within each community type, we constructed correlation networks based on Spearman correlation coefficients (P value <0.001) within each community type [31]. The dominant genus within community types I, III and IV was negatively correlated with less abundant community members (Figure 4A, C, D). The correlation network for the high diversity community type II was sparser, suggesting that changes in the relative abundance of specific taxa occur independently (Figure 4B).

### Host Genetic Diversity and the Fecal Microbiota

**Figure 5:**
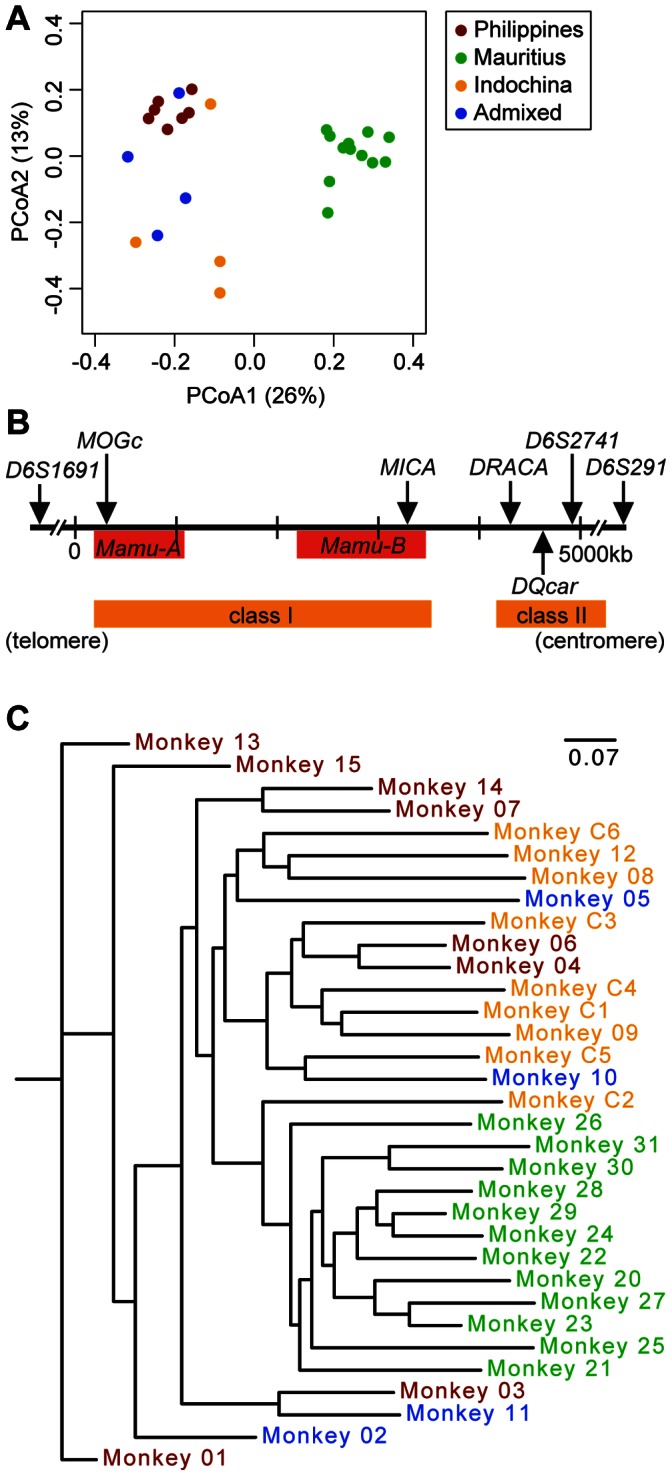
Analysis of genetic variability of cynomolgus macaques in studies 1–4. (**A**) Principal coordinates analysis (PCoA) based on Lynch pairwise distance calculated from 24 STR data derived from archived peripheral blood lymphocytes from each cynomolgus macaque in studies 1, 2, and 3. Each individual dot represents an individual macaque, color-coded by geographic origin (boxed inset), with percent variance shown on each axis. (**B**) Localization of seven microsatellite markers tested within the MHC region (adapted from rhesus macaque MHC map) [26]. (**C**) Phylogenetic tree calculated from indicated MHC microsatellite data for all macaques (studies 1–4), using the Lynch pairwise distance measure. Individual macaques are color-coded as indicated in the boxed inset in (**A**).

Several reports have revealed that cynomolgus macaques obtained from different geographic origin represent different genetic backgrounds, including differences within the major histocompatibility complex (MHC) [26], [32], [33]. To confirm the geographic origin of macaques in these studies, genotype analysis on 24 non-MHC, short tandem repeats (STR) from archived peripheral blood lymphocytes from cynomolgus macaques in studies 1, 2, and 3 was performed by the Molecular Anthropology Laboratory (Davis, CA) and Primate Products, Inc., Miami, FL (Materials and Methods). Results were compared to reference STR data obtained from known Sumatran (Indonesian), Mauritian, Philippine and Vietnamese macaques [34]. The data revealed that the macaques in these studies originated from Indochina, Philippines, Indonesia or Mauritius (Table S1). PCoA of the STR data indicated separation of Mauritius macaques (study 2) from macaques of other geographic origin (Figure 5A). No clustering was identified among the other populations. Genotype was not analyzed for study 4 macaques (n = 6); however, according to the vendor (Harlan Laboratories, Inc.; Indianapolis, IN), these macaques originated from Indochina. Each study displayed a different distribution of genotypes. Study 1 macaques were of Indochinese, Philippine and/or Indonesian origin (n = 12), all study 2 macaques were of Mauritian origin (n = 12), and all study 3 macaques were of Philippine origin (n = 3). Combining the data regarding composition of the fecal microbiota with the STR genotype analysis, we observed that cynomolgus macaques from different geographic origins harbored different communities of fecal microbiota. Most notably, the fecal microbiota in the majority of macaques from Mauritius (study 2) represent the high-diversity community type II, characterized by an abundance of taxa from both the Firmicutes and the Bacteroidetes and a significantly lower level of *Lactobacillus* as compared to the other community types (Figures 2–4 and Table 1). The fecal microbiota in macaques from Indochina, Indonesia, and the Philippines were less diverse, and contained low levels of any genera from the phylum Bacteroidetes.

Previous studies have reported that differences in cynomolgus macaque MHC haplotypes are associated with differential susceptibility to and resolution of infectious diseases [35], [36]. Cynomolgus macaques originating from Mauritius have been reported to exhibit a more restricted MHC allele repertoire compared to those from Indochina, Indonesia or Vietnam [32], [33], [37], [38]. To assess the genetic differences among our cynomolgus macaque population, we genotyped seven previously reported MHC microsatellites spanning both class I and class II regions: *D6S1691, MOGc, MICA, DRACA, DQcar, D6S2741,* and *D6S291* [26], [39] (Figure 5B) (Materials and Methods). The allele frequencies for each geographically distinct population (Philippines, Mauritius, Indochina or Admixed) within each loci indicated a broad range of MHC allele types within each population (Figure S2). A phylogenetic tree calculated using the Lynch distance from the MHC microsatellite data indicated distinct clustering of all macaques originating from Mauritius (Figure 5C). Using ANOVA, we identified significant correlations to at least one community type within four of the seven loci (*D6S1691, D6S2741, DQCar,* and *DRACA*), suggesting that allelic differences in these loci may contribute to community type structure (p<0.01, Table S2). These results confirm that divergent MHC allele repertoires are represented in geographically distinct macaques and suggest that differences in the intestinal microbiota in cynomolgus macaques may correlate with host genetic differences, including those found in MHC alleles.

### Impact of Live-attenuated and Wild-type *S. dysenteriae* 1 Strains on Microbiota Composition

Longitudinal fecal sampling was conducted for all macaques in each study (Figure 1). To assess the stability of the fecal microbiota in cynomolgus macaques, we compared the Shannon diversity index and community type variation over time, as well as the impact of immunization and wild-type challenge on community composition. In macaques from studies 1 and 2, we assessed the efficacy of two live-attenuated *S. dysenteriae* 1 vaccine candidates, CVD1255 and CVD1256 [18], to protect against subsequent challenge with wild-type *S. dysenteriae* 1 strain 1617 using macaques that received phosphate buffered saline (PBS) as controls. Macaques in study 3 were only challenged with wild-type *S. dysenteriae* 1 strain 1617. Macaques in study 4 received no treatment throughout the course of sample collection. All groups were monitored on a daily basis for development of clinical signs of shigellosis and serum was collected to measure IgA and IgG antibody titers against *S. dysenteriae* 1 LPS.

**Figure 6:**
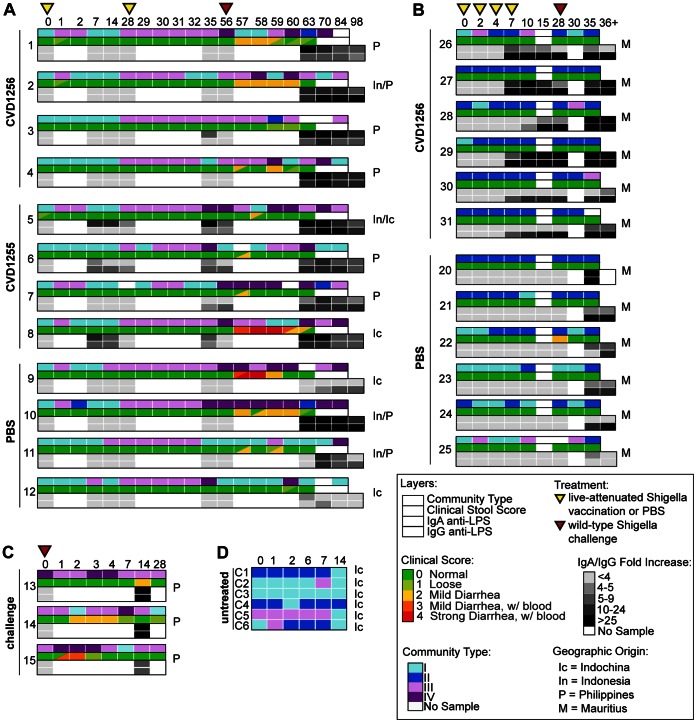
Effect of *Shigella* strains on the fecal microbiota in cynomolgus macaques. Community type composition, clinical disease symptoms, and elicited immune response over time for (**A**) study 1, (**B**) study 2, (**C**), study 3, and (**D**) study 4. Individual macaque identification and treatment administered (CVD1255, CVD1256 or PBS) are indicated on the y-axis (left), with geographic origin of each macaque on the right. Sampling day is indicated on the x-axis, with color-coded arrows (immunization or challenge) indicating time of treatment (boxed inset). Community type (**A**–**D**) is indicated on the first top bar, stool severity score ranging from 1 (mild) to 4 (severe) (**A** to **C**) is indicated on the second bar, and anti-LPS IgA and anti-LPS IgG fold differences over antibody levels before immunization and challenge (day 0) (**A**–**C**) are indicated on the third and fourth (bottom) bars, respectively, each color-coded according to the boxed inset, with day 0 antibody titers shown the same as <4 fold increases.

As expected, the fecal microbiota in the control group (study 4, no intervention), was stable over a period of 14 days as evidenced by community type persistence (Figure 6D). In the absence of intervention, relatively few community type changes occurred within an individual macaque. With the exception of macaque C5, both the high diversity community type II and the moderately diverse community type I were found. A similar Shannon diversity index (Figure S3) further confirmed community stability over time within untreated macaques.

Community stability over time was also high in all samples taken from macaques in study 2, despite the fact that these animals received four immunizations with CVD1256, or mock immunization with PBS over a seven-day period (Figure 6B). More than 90% of the macaques in study 2 harbored the high diversity community type II at one or more times points. Little or no change in community type or the Shannon diversity index was observed following administration of PBS or CVD1256 and subsequent wild-type challenge (Figure 6B and S3). Furthermore, using the Wilcoxon nonparametric test, no significant differences were observed in the Shannon diversity index for pre-treatment, post-vaccination or post-challenge samples when compared to each other or to study 4 samples (Figure S4).

Greater variation was observed across time in fecal samples obtained from macaques in study 1 (two doses of live-attenuated *Shigella* vaccines followed by wild-type challenge). The fecal microbiota of the macaques in study 1 was initially represented by community type I, which is dominated by both *Streptococcus* and *Lactobacillus* (Figure 6A). Following the first treatment (CVD1255, CVD1256 or PBS administration), there was a shift in structure of the microbiota in 58% of the macaques to the lower diversity community type III, dominated by *Lactobacillus*. Following the second immunization (day 28), the microbiota in all macaques shifted to community type III. The Shannon diversity index for study 1 samples within both immunized and PBS-administered macaques changed as a result of treatment (Figure S3), and the Wilcoxon test indicated that the diversity was significantly reduced following these two treatments compared to pre-treatment time points (day 0) or samples from the untreated study 4 macaques (Figure S4). Changes in community composition were observed in all macaques, even those receiving PBS, suggesting that the immunization protocol (i.e., anesthesia and animal handling), rather than immunization with attenuated strains of *Shigella*, was responsible for the observed changes.

Subsequent challenge with wild-type *S. dysenteriae* 1 in study 1 (day 56) resulted in a shift to the *Enterococcus*-dominated community type IV at one or more time points, in 91% of the macaques (Figure 6A). In fecal samples collected within two days post-challenge, we also observed an increase in the proportion of low abundance genera, including *Pedobacter, Flavobacterium, Comamonas*, and *Yersinia*, (Figure S5 and Dataset S1). In post-challenge samples from 40% of the macaques, the relative abundance of these less prevalent genera was as high as 80% (Dataset S1). We also observed an increase in the Shannon diversity index in post-challenge samples (Figure S3, S4), which likely reflects the increase in the relative abundance of these rarer genera.

Stool consistency was monitored in studies 1–3 for signs of clinical shigellosis. A stool score ranging from 1 (loose stool) to 4 (severe bloody diarrhea) was recorded using previously established measures (Materials and Methods) [10]. No macaques experienced severe symptoms of shigellosis following vaccination (<2 days post-vaccination), but 83% of the macaques in study 1 exhibited clinical symptoms of *S. dysenteriae* 1 infection (stool score of grades 2 or higher at one or more time points) following wild-type challenge (Figure 6A). Using the Wilcoxon test, a significant correlation was observed between stool scores of 2 or more and an increase in the abundance of *Yersinia, Comamonas, Pedobacter*, and *Flavobacterium* (Figure S5). No macaques in study 2, including those receiving PBS prior to challenge, experienced clinical disease symptoms associated with wild-type challenge. Moreover, there were no detectable increases in the relative abundance of rare genera in any of these samples, consistent with the observation of community resistance to perturbation from both treatment and pathogen infection (Figure 6B and Dataset S1). *Shigella* sequences were not identified by 16 S rRNA profiling following challenge or immunization despite protocol confirmation of the strains using culture methods 1–3 days post-immunization and post-challenge.

In study 3, unvaccinated macaques were challenged on day 0 with wild-type *S. dysenteriae* 1. As observed in study 1, the composition of the fecal microbiota was altered following exposure to wild-type *Shigella*, and the *Enterococcus*-dominated community type IV emerged (Figure 6C). Clinical symptoms of shigellosis were observed in 2 of the 3 macaques in this study.

In contrast to the clinical symptomology, all challenged macaques (studies 1–3) mounted *Shigella*-specific immune responses following administration of wild-type *S. dysenteriae* 1, as measured by IgA and IgG antibodies against *S. dysenteriae* 1 LPS (Figure 6A–C). All immunized macaques in studies 1 and 2 (macaques 1–8, 20–25) mounted a *Shigella*-specific immune response following immunization (Figure 6A, B). Compared to immunized macaques, macaques receiving PBS prior to challenge in both studies (macaques 9–12, 26–31) elicited a less robust immune response post-challenge, despite the lack of clinical signs of shigellosis in study 2 macaques (Figure 6A, B).

To investigate whether specific members of the microbiota were significantly correlated with serum antibody responses or clinical symptoms, we implemented a statistical tool called local similarity analysis (LSA) [24] to identify time-dependent correlations. Compared to Pearson correlation analysis, which is typically utilized to identify linear relationships, the LSA ecological time-series model is able to identify complex, time-dependent interactions among microbial members as well as various environmental parameters. From the LSA, we then constructed a network for each study using only significant associations calculated by this method (LSA correlation coefficient >0.4, p<0.01) (Dataset S2).

**Figure 7:**
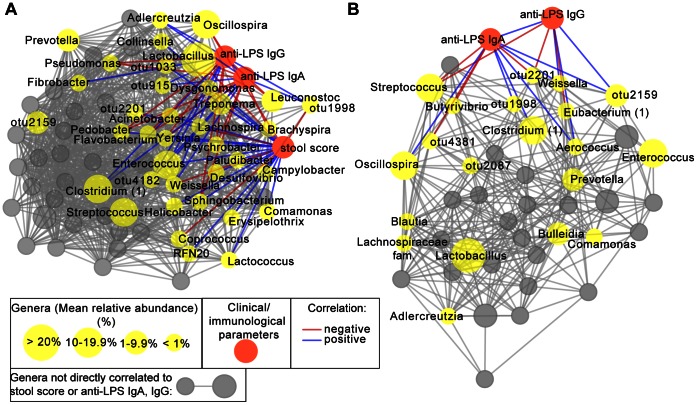
Local similarity analysis between genera and clinical and/or immunological measurements in macaque *Shigella* vaccine studies. Networks of significant correlations (p<0.01; q >0.40) using local similarity analysis between genera and the immunological or clinical measurements in macaques in (**A**) study 1 and (**B**) study 2. Nodes are colored as described in boxed inset (genera sized for relative abundance). Correlations among non-core genera and genera not directly related to immunological or clinical measurements have been greyed out.

Due to the different collection periods, vaccine regimen, and significant differences in community structure of the microbiota observed in the two studies, it is difficult to directly compare the antibody responses and microbiota between the studies 1 and 2. However, within-study associations revealed associations between the microbiota and immunological parameters. The network for study 1 was dense, suggesting a complex relationship among genera observed in the microbiota, antibody response, and clinical stool score (Figure 7A). The clinical stool score was positively associated with many genera, including the genera *Flavobacterium, Pedobacter, Yersinia,* and *Enterococcus*, further substantiating our observations of increased abundance of rare genera following challenge. Network analysis in study 1 also revealed increases in other rare genera in some monkeys following challenge, including *Psychrobacter, Erysipelothrix, Paludibacter*, and *Dysgomonas* (Dataset S1).

Compared to study 1, the resulting network for study 2 was less dense despite a robust antibody response following challenge. This is likely a reflection of the community stability observed over time in these macaques despite multiple immunizations and subsequent challenge. In study 2, anti-*Shigella* LPS antibody responses correlated with many members of the core microbiota, including positive correlations with core members such as *Oscillospira,* otu2159, and otu1998, but a negative correlation with *Streptococcus*. Interestingly, both studies shared positive correlations between anti-*Shigella* LPS antibody response and *Weissella*, otu1998, and *Clostridium* (clade 1). These results demonstrate that while differences in microbiota composition did not impair the host’s ability to mount IgA and IgG anti-LPS antibody responses following intragastric immunization or challenge, the susceptibility to disease following wild-type challenge was still markedly different between macaque populations of different geographic origin, which also exhibited distinct differences in diversity measures and composition.

## Discussion

In this study we used 16 S rRNA pyrosequencing to define community types that characterize the fecal microbiota of cynomolgus macaques and characterized community dynamics in response to immunization and wild-type challenge. Compared to the fecal microbiota in humans, the fecal microbiota in cynomolgus macaques is dominated by Firmicutes, particularly the genus *Lactobacillus* [23]. In the absence of immunization or challenge, the microbial community remains stable over time, as observed in the untreated control group of macaques (study 4). We observed two distinct community types in healthy macaques, suggesting that enterotypes may also exist in non-human primates; however, confirmation of this will require examination of a larger number of animals.

The inoculum dose to trigger shigellosis in NHPs is higher than that for humans; this has been attributed differences in gut physiology and microbiota [1]. In this study, we observed a differential response to *S. dysenteriae* 1 immunization and subsequent wild-type challenge for macaques of Mauritian origin compared to those of Indonesian/Indochinese/Philippine origin. Mauritian macaques harbored a high-diversity community type II, which was stable compared to other community types following immunization and challenge. Additionally, Mauritian macaques did not develop any clinical shigellosis symptoms following challenge with wild-type *S. dysenteriae* 1 (study 2). These results contrast with the significant changes in diversity, community type, and clinical outcome observed in macaques from Indochina/Indonesia/Philippines. Taken together, these findings suggest that a more diverse intestinal microbiota may play a protective role in response to enteric pathogens. This is consistent with ecological stability-diversity theory, which suggests that higher diversity within an ecosystem confers stability in response to environmental perturbations [40], [41]. Although environmental factors may in part shape these communities, all macaques were subjected to a three-month quarantine period prior to study collection, presumably decreasing or eliminating the impact of the conditions in which different vendors keep the animals or diet origins. It is notable that within the clinically susceptible macaques (study 1), changes in the diversity of the intestinal microbiota were seen in macaques receiving either PBS or live-attenuated bacteria, suggesting that the protocol for immunization, which involved handling, anesthesia, and endoscopy, was responsible for the observed changes. Both human and animal studies have shown that stress can reduce gastrointestinal microbial diversity [42]–[44] and affect immune function [45], [46], possibly leading to increased susceptibility to pathogen infection. Of note, changes in the microbiota were not observed in the non-susceptible macaques (study 2) receiving either PBS or live-attenuated bacteria, further supporting the observation that the microbiota in these macaques was less sensitive to perturbation in response to the interventions described in these studies. While an exhaustive analysis of host genetic factors that could potentially influence the outcome of these immunization studies was not carried out, future studies directed to investigate whether a protective microbiota can be transferred between different cynomolgus macaque populations, or the effects of antibiotic treatment on susceptibility, will provide additional insights into the potential role of the microbiota in protection against infection with enteric pathogens.

Together, these data highlight the importance of considering host genotype, environmental factors, and the resident gut microbiota in the context of vaccine response. Several murine models have implicated host genotype as a determinant of microbiota composition. Previous studies suggest that due to the close interaction of the immune system with the gastrointestinal environment, the highly polymorphic major histocompatibility complex (MHC) alleles may contribute to the observed variability of the microbiota within the host [47]. Our genotype analysis of seven microsatellites spanning the MHC region supports previous observations that the MHC alleles within cynomolgus macaques vary depending on their geographic origin. In this study, Mauritian cynomolgus macaques were more resistant to *S. dysenteriae* 1 infection than other macaques and also harbored a more diverse gut microbiota. While the impact of distinct MHC alleles on the intestinal microbiota has not been reported, our data suggests a possible role for host genetics, including MHC variability, in shaping this microbial community.

Studies evaluating differences in microbiota composition in relation to vaccine efficacy in various human populations worldwide are lacking. Similarly, very little information is available regarding associations between defined HLA haplotypes and response to vaccination in humans. Notably, individuals with certain HLA haplotypes have been reported to exhibit reduced responsiveness to hepatitis B vaccination [48]. NHP populations may be useful in assessing immune responses in initial phases of vaccine studies, but may not be fully predictive of the responses in a highly diverse human population. If distinct microbiota composition is responsible, at least in part, for differences in susceptibility to infection with enteric pathogens as our studies in cynomolgus macaques suggest, research on the global variation in the human microbiota composition may be important in the interpretation of the results from field studies involving immunization with vaccine candidates against *Shigella* and other enteric diseases such as *Salmonella enterica* serovar Typhi, *S.* Paratyphi A and B, and rotavirus.

## Supporting Information

Figure S1 **Top 20 genera within cynomolgus macaque studies.** Rank abundance plots of 20 most abundant genera for (**A**) study 1, (**B**) study 2, (**C**) study 3, and (**D**) study 4. Relative read abundance (within total reads per study) is shown on the y-axis, and genera are listed on the x-axis.
(TIF)

Figure S2 **Allele frequencies for different geographic populations for seven MHC loci.** Allele identity (**A** to **G**) and its frequency per geographic population are indicated on the y-axis, and the nucleotide length of microsatellite alleles on the x-axis. Frequencies are color-coded by geographic population (boxed inset).
(TIF)

Figure S3 **Shannon diversity index over time by study group.** Boxplots of the median shannon diversity index over time for samples from all macaques in (**A**) study 1 (n = 12), (**B**) study 2 (n = 12), (**C**) study 3 (n = 3), and (**D**) study 4 (n = 6). The Shannon diversity index is indicated on the y-axis, and time (in days) on the x-axis. Error bars within boxplots indicate the interquartile range between the first and third quartiles. Yellow arrows indicate treatment with either live-attenuated vaccine strains or PBS, and red arrows indicate challenge with wild-type *S. dysentariae* 1 as indicated in the boxed inset.
(TIF)

Figure S4 **Changes in the Shannon diversity index following immunization or PBS administration and wild-type challenge in all macaques from studies 1 and 2 compared to control study 4 macaques.** (**A**) Study 2 (n = 12): Boxplots of median Shannon diversity of pre-treatment time period samples (day 0), post-treatment samples (days 2–28, CVD1256 or PBS), and post-challenge samples (days 30–35, wild-type *S. dysenteriae* 1) from all macaques in study 2 (n = 12) compared to samples from control study 4 macaques (n = 6). (**B**) Study 1: Boxplots of median Shannon diversity index of pre-treatment time period samples (day 0), post-1^st^-treatment samples (days 1–28, CVD1255, CVD1256, or PBS), post-2^nd^-treatment samples (days 29–56, 2^nd^ dose of same treatment), post-challenge samples (days 57–84) for all macaques (n = 12) in study 1 compared to samples from control study 4 macaques (n = 6). The nonparametric wilcoxon test was used for all statistics, and error bars indicate the interquartile range between the first and third quartiles.
(TIF)

Figure S5 **Increase in the relative abundance of normally rare organisms correlates with clinical severity. (A)** Increase in relative abundance of normally rare genera over time in study 1 macaques (n = 12). Relative read abundance is on the y-axis and time (days) on the x-axis. Each point represents an individual sample and is color-coded as indicated in the boxed inset. **(B)** Relative read abundance of less abundant organisms and clinical symptoms of *Shigella* infection in study 1 macaques (n = 12). The relative read abundance for *Pedobacter, Yersinia, Flavobacterium*, and *Comamonas* (y-axis) over time in days (x-axis), color-coded by clinical score of stool symptom severity. Description of severity is listed in boxed inset. Significant correlations were calculated using a one-way nonparametric Wilcoxon test, comparing the indicated genus abundance in stool specimens with a clinical stool score of ≥2 compared to a null clinical score of 0.
(TIF)

Table S1 **Probability of geographic origin for cynomolgus macaques.** Probabilities based on STR data genotypes analyzed in conjunction with known Sumatran (Indonesian), Mauritian, Philippine and Vietnamese (Indochinese) cynomolgus macaques (n = 27).
(DOC)

Table S2 **Correlation of microsatellite regions to community type persistence using ANOVA.** For each macaque, microsatellite alleles and community type relative abundance (measured as percent time spent in each community type) were analyzed using ANOVA. Microsatellite alleles that occurred less than three times were excluded, and homozygous alleles were counted as one. Community type relative abundance was normalized using arcsin(sqrt()) transformation. Significant correlations between microsatellite regions and community types (p-value <0.01) are indicated by the symbol (*).
(DOC)

Text S1 Supporting Methods.
(DOC)

Dataset S1 **Metadata and sequence information for cynomolgus macaques within live-attenuated** ***S. dysenteriae*** **1 study groups (1–4): sample metadata and community composition (percentage of Greengenes database assignments) for each sample (n = 374).**
(XLS)

Dataset S2 **List of significantly correlated genera and immunological or clinical measurements (p<0.01; LSA Q value >0.40) calculated using local similarity analysis (LSA) for macaques in study 1 and 2.**
(XLS)
