# Supplementary figures and images for: Differential Response of the Cynomolgus Macaque Gut Microbiota to Shigella Infection
Source: PLoS One. 2013 Jun 5;8(6):e64212. doi: 10.1371/journal.pone.0064212 (PMC3673915; doi:10.1371/journal.pone.0064212)

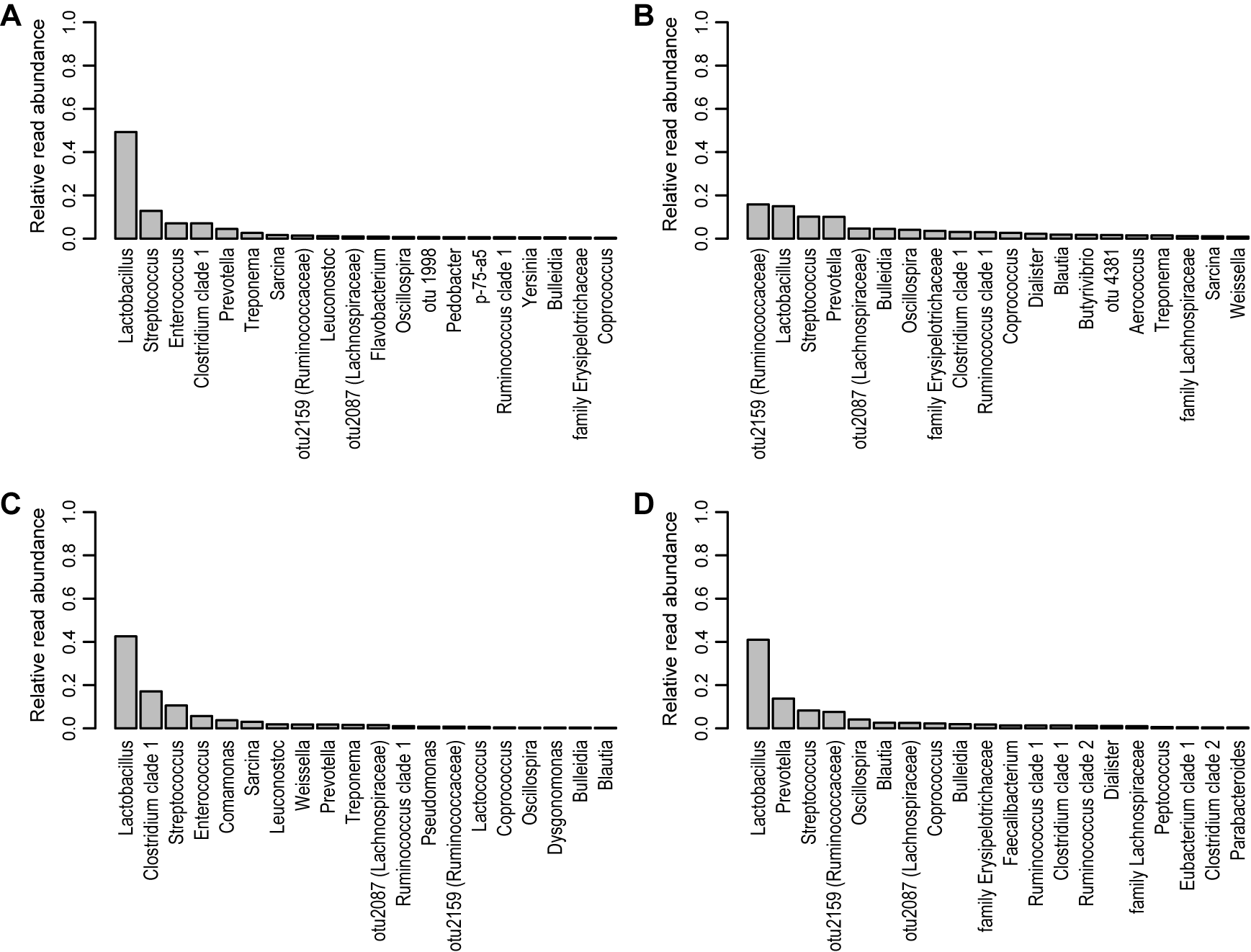

Supplement: Figure S1 — Top 20 genera within cynomolgus macaque studies. Rank abundance plots of 20 most abundant genera for (A) study 1, (B) study 2, (C) study 3, and (D) study 4. Relative read abundance (within total reads per study) is shown on the y-axis, and genera are listed on the x-axis. (TIF) [file pone.0064212.s001.tif]

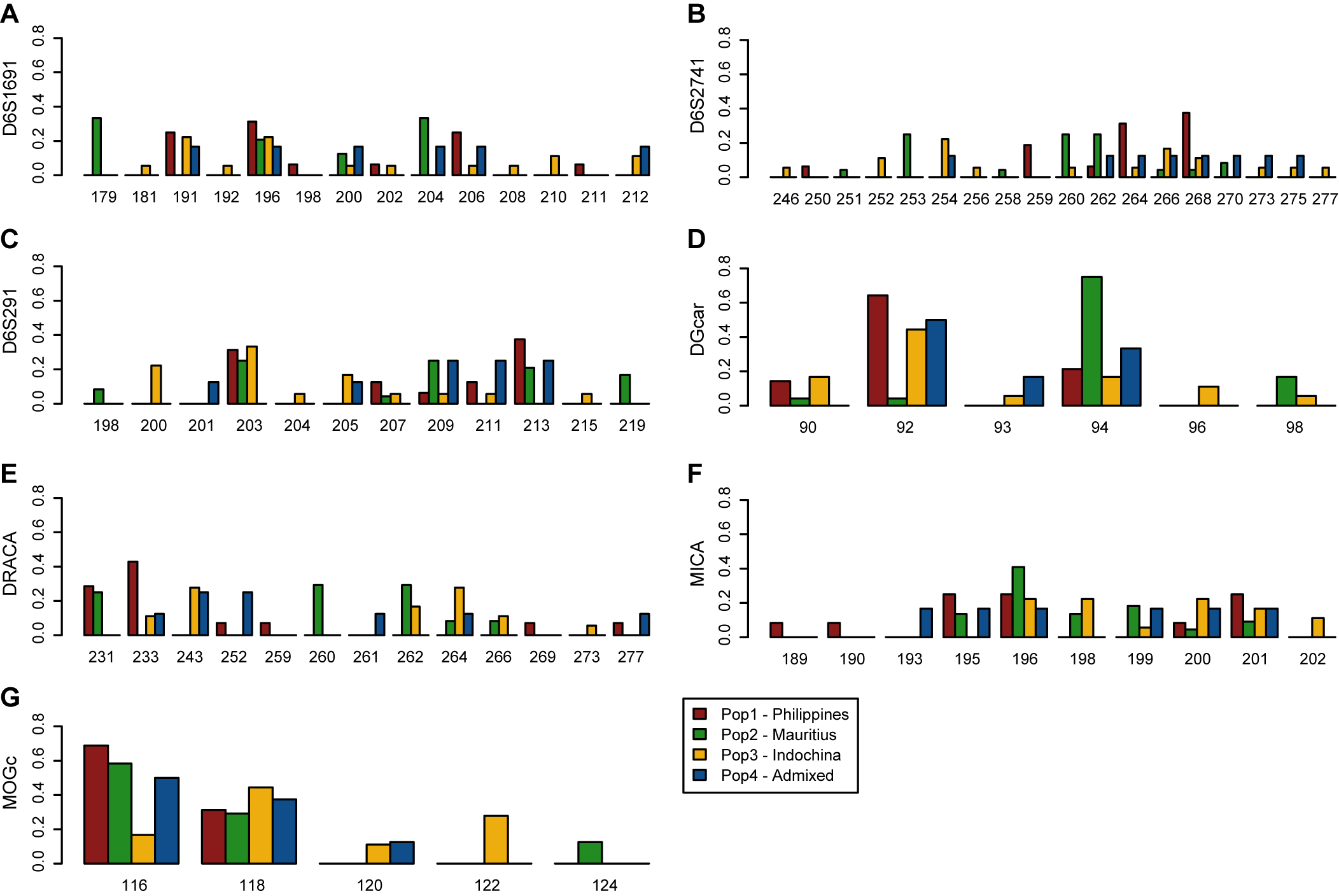

Supplement: Figure S2 — Allele frequencies for different geographic populations for seven MHC loci. Allele identity (A to G) and its frequency per geographic population are indicated on the y-axis, and the nucleotide length of microsatellite alleles on the x-axis. Frequencies are color-coded by geographic population (boxed inset). (TIF) [file pone.0064212.s002.tif]

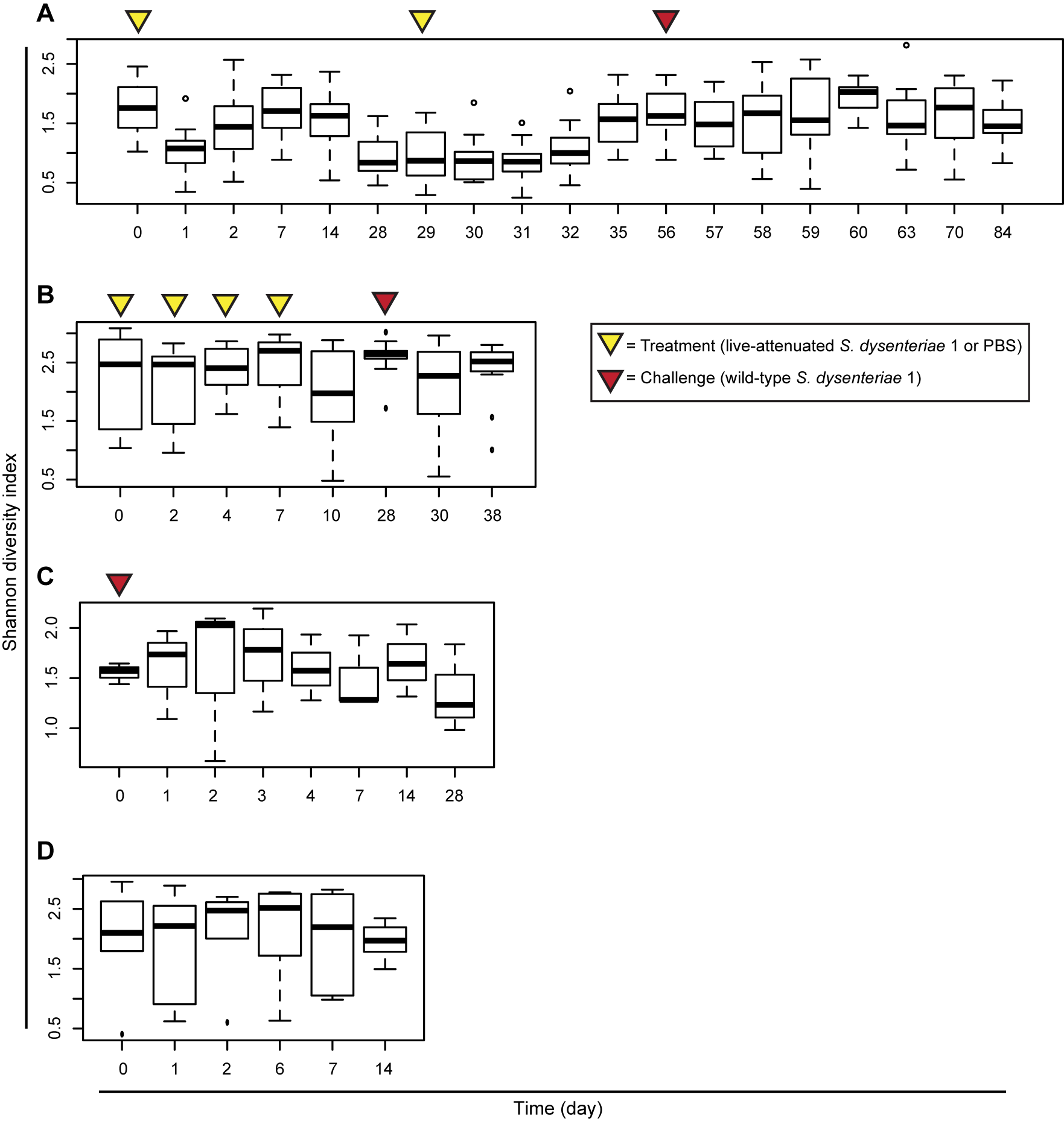

Supplement: Figure S3 — Shannon diversity index over time by study group. Boxplots of the median shannon diversity index over time for samples from all macaques in (A) study 1 (n = 12), (B) study 2 (n = 12), (C) study 3 (n = 3), and (D) study 4 (n = 6). The Shannon diversity index is indicated on the y-axis, and time (in days) on the x-axis. Error bars within boxplots indicate the interquartile range between the first and third quartiles. Yellow arrows indicate treatment with either live-attenuated vaccine strains or PBS, and red arrows indicate challenge with wild-type S. dysentariae 1 as indicated in the boxed inset. (TIF) [file pone.0064212.s003.tif]

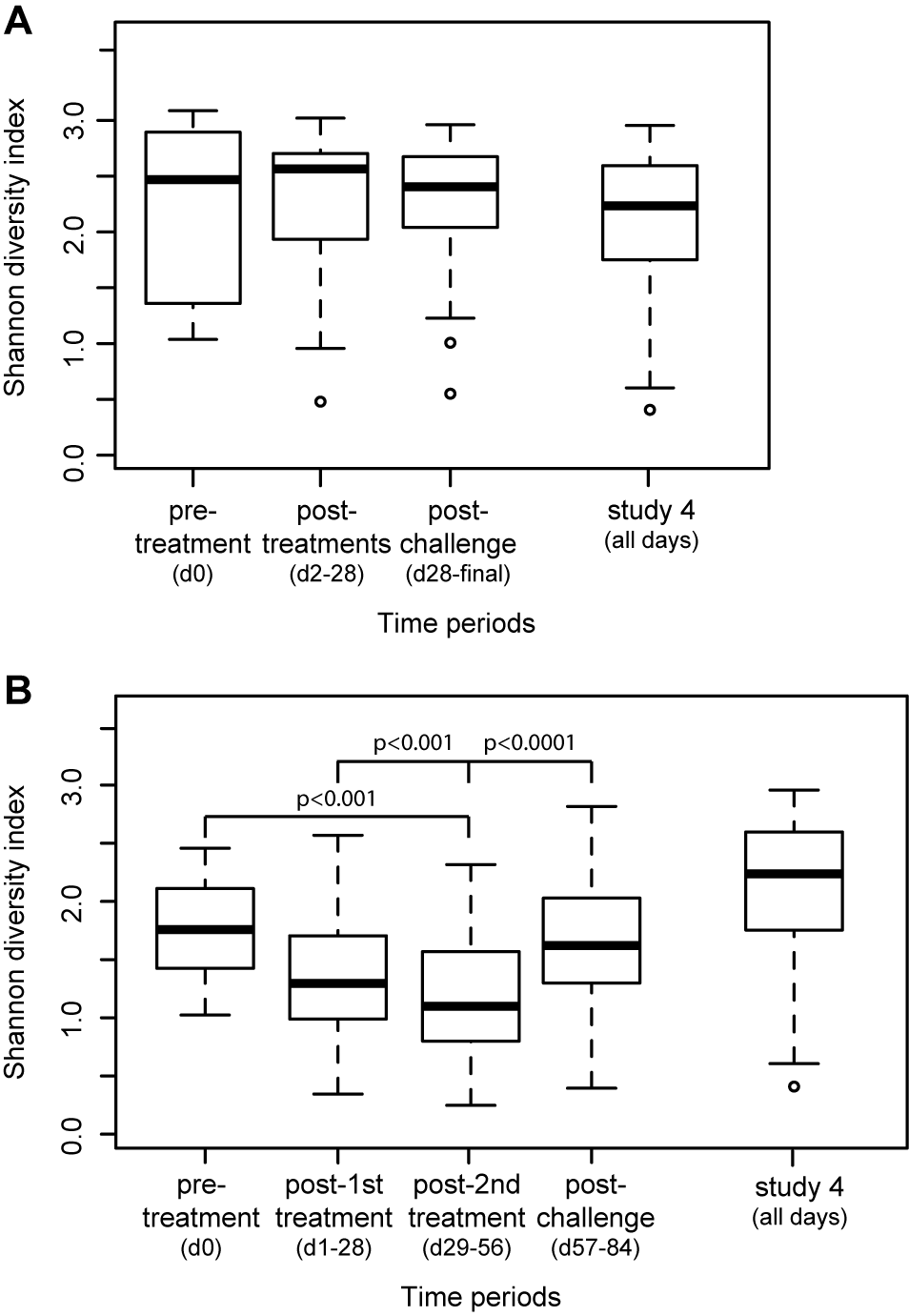

Supplement: Figure S4 — Changes in the Shannon diversity index following immunization or PBS administration and wild-type challenge in all macaques from studies 1 and 2 compared to control study 4 macaques. (A) Study 2 (n = 12): Boxplots of median Shannon diversity of pre-treatment time period samples (day 0), post-treatment samples (days 2–28, CVD1256 or PBS), and post-challenge samples (days 30–35, wild-type S. dysenteriae 1) from all macaques in study 2 (n = 12) compared to samples from control study 4 macaques (n = 6). (B) Study 1: Boxplots of median Shannon diversity index of pre-treatment time period samples (day 0), post-1st-treatment samples (days 1–28, CVD1255, CVD1256, or PBS), post-2nd-treatment samples (days 29–56, 2nd dose of same treatment), post-challenge samples (days 57–84) for all macaques (n = 12) in study 1 compared to samples from control study 4 macaques (n = 6). The nonparametric wilcoxon test was used for all statistics, and error bars indicate the interquartile range between the first and third quartiles. (TIF) [file pone.0064212.s004.tif]

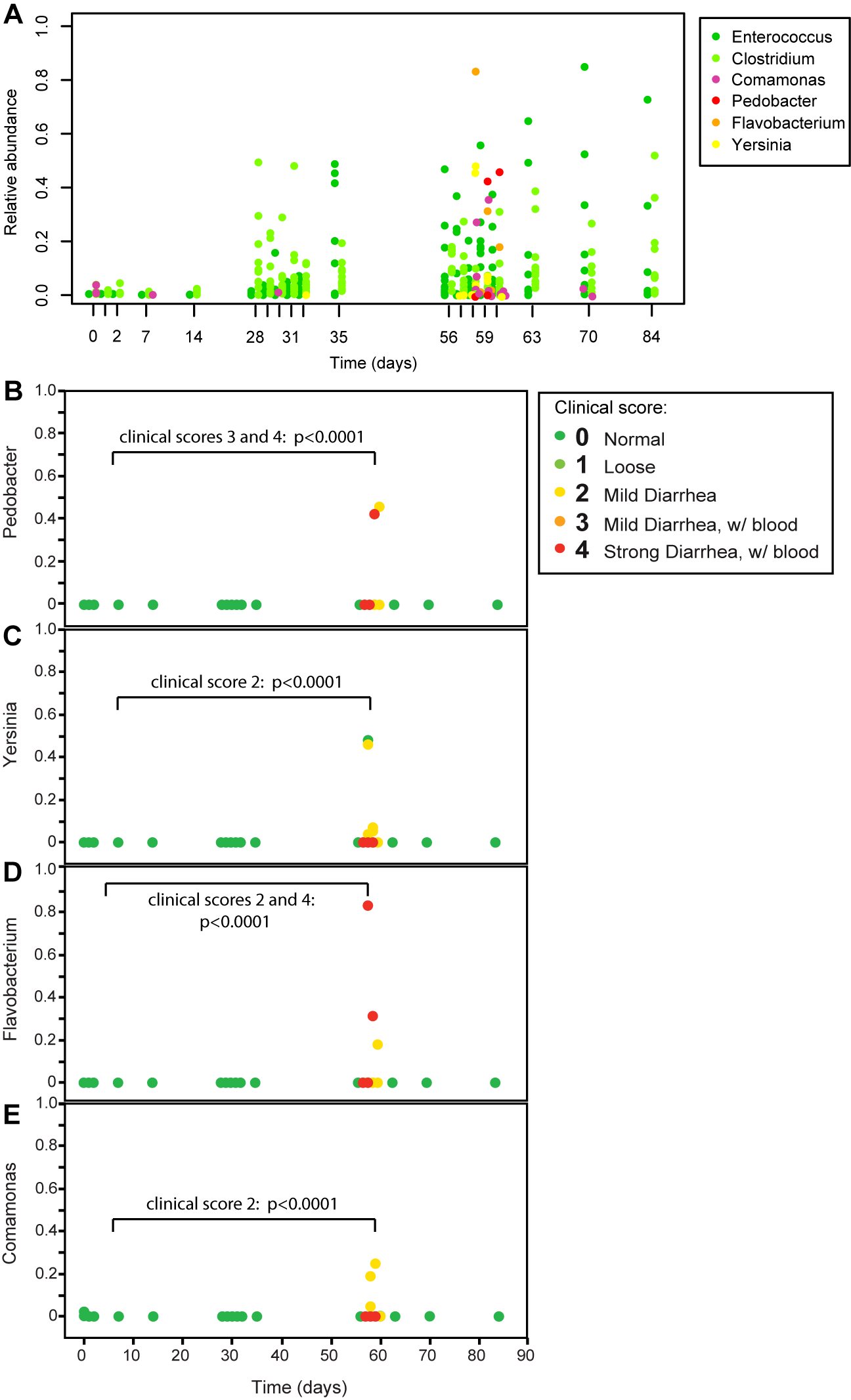

Supplement: Figure S5 — Increase in the relative abundance of normally rare organisms correlates with clinical severity. (A) Increase in relative abundance of normally rare genera over time in study 1 macaques (n = 12). Relative read abundance is on the y-axis and time (days) on the x-axis. Each point represents an individual sample and is color-coded as indicated in the boxed inset. (B) Relative read abundance of less abundant organisms and clinical symptoms of Shigella infection in study 1 macaques (n = 12). The relative read abundance for Pedobacter, Yersinia, Flavobacterium, and Comamonas (y-axis) over time in days (x-axis), color-coded by clinical score of stool symptom severity. Description of severity is listed in boxed inset. Significant correlations were calculated using a one-way nonparametric Wilcoxon test, comparing the indicated genus abundance in stool specimens with a clinical stool score of ≥2 compared to a null clinical score of 0. (TIF) [file pone.0064212.s005.tif]
