# Supplementary material for: Differential Response of the Cynomolgus Macaque Gut Microbiota to Shigella Infection
Source: PLoS One. 2013 Jun 5;8(6):e64212. doi: 10.1371/journal.pone.0064212 (PMC3673915; doi:10.1371/journal.pone.0064212)
Supplement: Table S1 — Probability of geographic origin for cynomolgus macaques. Probabilities based on STR data genotypes analyzed in conjunction with known Sumatran (Indonesian), Mauritian, Philippine and Vietnamese (Indochinese) cynomolgus macaques (n = 27). (DOC) [file pone.0064212.s006.doc]

**Table S2. Probability of geographic origin for cynomolgus macaques.**

| - **Macaque** | - **Study** | - **Probability Mauritian Origin** | - **Probability Philippine Origin** | - **Probability Indochinese Origin** | - **Probability Indonesian Origin** | - **Most likely assignment** |
| --- | --- | --- | --- | --- | --- | --- |
| - 1 | - 1 | - 0.0000 | - 0.9990 | - 0.0000 | - 0.0010 | - Philippines |
| - 2 | - 1 | - 0.0000 | - 0.3499 | - 0.0686 | - 0.5814 | - Admixed (Philippines, Indonesia) |
| - 3 | - 1 | - 0.0000 | - 1.0000 | - 0.0000 | - 0.0000 | - Philippines |
| - 4 | - 1 | - 0.0000 | - 0.9998 | - 0.0000 | - 0.0002 | - Philippines |
| - 5 | - 1 | - 0.0000 | - 0.0205 | - 0.4712 | - 0.5083 | - Admixed (Indochina, Indonesia) |
| - 6 | - 1 | - 0.0000 | - 1.0000 | - 0.0000 | - 0.0000 | - Philippines |
| - 7 | - 1 | - 0.0000 | - 0.8609 | - 0.0012 | - 0.1380 | - Philippines |
| - 8 | - 1 | - 0.0000 | - 0.0000 | - 0.9509 | - 0.0491 | - Indochina |
| - 9 | - 1 | - 0.0000 | - 0.0000 | - 0.9979 | - 0.0021 | - Indochina |
| - 10 | - 1 | - 0.0000 | - 0.6457 | - 0.0834 | - 0.2708 | - Admixed (Philippines, Indonesia) |
| - 11 | - 1 | - 0.0000 | - 0.5810 | - 0.0002 | - 0.4188 | - Admixed (Philippines, Indonesia) |
| - 12 | - 1 | - 0.0000 | - 0.0538 | - 0.9251 | - 0.0211 | - Indochina |
| - 13 | - 3 | - 0.0000 | - 0.9010 | - 0.0002 | - 0.0988 | - Philippines |
| - 14 | - 3 | - 0.0000 | - 0.9981 | - 0.0000 | - 0.0019 | - Philippines |
| - 15 | - 3 | - 0.0000 | - 1.0000 | - 0.0000 | - 0.0000 | - Philippines |
| - 20 | - 2 | - 1.0000 | - 0.0000 | - 0.0000 | - 0.0000 | - Mauritius |
| - 21 | - 2 | - 1.0000 | - 0.0000 | - 0.0000 | - 0.0000 | - Mauritius |
| - 22 | - 2 | - 1.0000 | - 0.0000 | - 0.0000 | - 0.0000 | - Mauritius |
| - 23 | - 2 | - 0.9968 | - 0.0000 | - 0.0000 | - 0.0032 | - Mauritius |
| - 24 | - 2 | - 0.9745 | - 0.0000 | - 0.0000 | - 0.0255 | - Mauritius |
| - 25 | - 2 | - 0.9987 | - 0.0000 | - 0.0000 | - 0.0013 | - Mauritius |
| - 26 | - 2 | - 1.0000 | - 0.0000 | - 0.0000 | - 0.0000 | - Mauritius |
| - 27 | - 2 | - 1.0000 | - 0.0000 | - 0.0000 | - 0.0000 | - Mauritius |
| - 28 | - 2 | - 0.9993 | - 0.0000 | - 0.0000 | - 0.0007 | - Mauritius |
| - 29 | - 2 | - 0.8695 | - 0.0001 | - 0.0000 | - 0.1303 | - Mauritius |
| - 30 | - 2 | - 1.0000 | - 0.0000 | - 0.0000 | - 0.0000 | - Mauritius |
| - 31 | - 2 | - 1.0000 | - 0.0000 | - 0.0000 | - 0.0000 | - Mauritius |
