# Supplementary material for: Differential Response of the Cynomolgus Macaque Gut Microbiota to Shigella Infection
Source: PLoS One. 2013 Jun 5;8(6):e64212. doi: 10.1371/journal.pone.0064212 (PMC3673915; doi:10.1371/journal.pone.0064212)
Supplement: Table S2 — Correlation of microsatellite regions to community type persistence using ANOVA. For each macaque, microsatellite alleles and community type relative abundance (measured as percent time spent in each community type) were analyzed using ANOVA. Microsatellite alleles that occurred less than three times were excluded, and homozygous alleles were counted as one. Community type relative abundance was normalized using arcsin(sqrt()) transformation. Significant correlations between microsatellite regions and community types (p-value <0.01) are indicated by the symbol (*). (DOC) [file pone.0064212.s007.doc]

**Table S3. Correlation of microsatellite regions to community type persistence using ANOVA.**

| **Microsatellite**  **Region** | **Community Type** | **p-value** | **Expected false positives (at p-value threshold)** | **False Discovery**  **Rate (FDR)** |
| --- | --- | --- | --- | --- |
| *D6S1691* | I | *0.039864 | 1.116186 | 0.079728 |
| *D6S1691* | II | *0.000039 | 0.001083 | 0.001083 |
| *D6S1691* | III | *0.012995 | 0.363858 | 0.033078 |
| *D6S1691* | IV | *0.020133 | 0.563722 | 0.043363 |
| *D6S2741* | I | 0.099996 | 2.799885 | 0.155549 |
| *D6S2741* | II | *0.002347 | 0.065721 | 0.01643 |
| *D6S2741* | III | *0.003078 | 0.086182 | 0.014364 |
| *D6S2741* | IV | *0.000906 | 0.025356 | 0.012678 |
| *D6S291* | I | 0.405476 | 11.353333 | 0.540635 |
| *D6S291* | II | 0.056623 | 1.585436 | 0.09909 |
| *D6S291* | III | 0.055153 | 1.544273 | 0.102952 |
| *D6S291* | IV | 0.123559 | 3.459648 | 0.182087 |
| *DQcar* | I | 0.585959 | 16.406862 | 0.631033 |
| *DQcar* | II | *0.002589 | 0.072489 | 0.014498 |
| *DQcar* | III | *0.005909 | 0.165464 | 0.023638 |
| *DQcar* | IV | *0.001791 | 0.050152 | 0.016717 |
| *DRACA* | I | 0.514623 | 14.409455 | 0.600394 |
| *DRACA* | II | *0.012601 | 0.352823 | 0.035282 |
| *DRACA* | III | *0.008104 | 0.22692 | 0.028365 |
| *DRACA* | IV | *0.009837 | 0.275439 | 0.030604 |
| *MICA* | I | 0.50677 | 14.189562 | 0.616937 |
| *MICA* | II | 0.071284 | 1.995955 | 0.117409 |
| *MICA* | III | *0.015767 | 0.441474 | 0.036789 |
| *MICA* | IV | 0.421681 | 11.807071 | 0.536685 |
| *MOGc* | I | 0.634246 | 17.758887 | 0.657737 |
| *MOGc* | II | 0.387102 | 10.838868 | 0.541943 |
| *MOGc* | III | 0.820853 | 22.983873 | 0.820853 |
| *MOGc* | IV | 0.53806 | 15.065684 | 0.602627 |
