# Supplementary material for: Differential Response of the Cynomolgus Macaque Gut Microbiota to Shigella Infection
Source: PLoS One. 2013 Jun 5;8(6):e64212. doi: 10.1371/journal.pone.0064212 (PMC3673915; doi:10.1371/journal.pone.0064212)
Supplement: Text S1 — Supporting Methods. (DOC) [file pone.0064212.s008.doc]

**Text S1. Supporting Methods.**

***Study design parameters***

Macaques in study 4 received no bacterial challenge throughout sample collection. Stool sample collections from this group were carried out during the quarantine period (91days) at the University of Maryland School of Medicine. For macaques in studies 1, 2 and 3, the optimal dose to induce shigellosis was established previously . The 27 macaques in these studies were fasted for 12 h prior to bacterial challenge. Animals were challenged intragastrically with 10^11^ CFU of the indicated *S. dysenteriae* 1 strain 1617. Each macaque received 15 mEq sodium bicarbonate intragastrically immediately prior to inoculation to prevent killing of the bacteria by gastric acid. Each animal was monitored twice daily for signs of shigellosis following challenge. Stool specimens were collected from each animal twice daily until 4 days after inoculation, as described for each study. Study 1: Animals (n=12) were given CVD1256 (n=4), CVD1255 (n=4), or phosphate buffered saline (PBS) (n=4) on days 0 and 28 (1 dose per animal). Each animal received wild-type *S. dysenteriae* 1 strain 1617 on day 56. Study 2: Animals (n=12) received CVD1255 (n=6) or PBS (n=6) on days 0, 2, 4, and 7 (4 doses per animal). Each animal received wild-type *S. dysenteriae* 1 strain 1617 on day 28. Study 3: Animals (n=3) received wild-type *S. dysenteriae* 1 strain 1617 on day 0.

***Animal geographic origin***

DNA extracted from archived peripheral blood lymphocytes were analyzed by the Molecular Anthropology Laboratory, (Davis, CA) and Primate Products, Inc. (Miami, FL) for geographic region of origin (studies 1-3, n=27). Twenty-four short tandem repeat (STR) genotypes were generated for each sample. The loci evaluated are: *D1s548, D2s1333, D3s1768, D4s1626, D5s1457, D6s501, D7s794, D7s1826, D8s1106, D8s1466, D9s921, D10s1432, D18s536, D8s1106, D9s921, D9s934, D11s2002, D11s1975, D13s318, D13s765, D18s537, D16s750, DXs2506*, and *AGAT007*. Twenty of the 24 loci listed above were used in a linear discriminate analysis to determine the most likely region of origin for each sample. Samples were analyzed in conjunction with 13 known Sumatran (Indonesian), 33 Mauritian, 78 Philippine and 20 Vietnamese longtail macaques to determine their probability of origin from these four regions . Probabilities of geographic origin are shown in Table S2.

***Preparation of the bacterial inocula***

Two vaccine candidates, CVD1255 (*S. dysenteriae* 1 strain 1617 *∆guaBA ∆sen ∆stxAB*) and CVD1256 (*S. dysenteriae* 1 strain 1617 s*∆guaBA ∆sen ∆stxA::mLpp-stxB*), were used in immunization studies 1 and 2. Live-attenuated *S. dysenteriae* 1 CVD1255 and CVD1256 and challenge inocula *S. dysenteriae* 1 strain 1617 were prepared from frozen master stocks, which were plated onto trypticase soy agar (Becton Dickinson, Franklin Lakes, NJ) containing 0.01% Congo red dye (Sigma Chemical, St Louis, MO). After an incubation period of 18 to 24 hours at 37 °C, *S. dysenteriae* 1 antiserum (Denka Seiken, Tokyo, Japan) was used to confirm the identity of single-well isolated Congo-red–positive colonies that exhibited *Shigella* morphologic characteristics, and picked and suspended in sterile saline. These colonies were used to inoculate trypticase soy agar plates for heavy growth, then incubated overnight at 37 °C. Overnight growth was harvested, washed, and standardized turbidmetrically, and appropriately diluted in PBS for inoculation. These inocula were used within 4 hours of preparation. To establish the actual inoculum that was administered to the cynomolgus macaques, diluted colony counts in PBS were performed in 2 to 3 replicate plates after overnight incubation.

***Intragastric inoculation of bacteria***

Macaques were sedated with 10 mg/kg ketamine IM prior to bacterial inoculation. After onset of sedation, an 8- to 14-French orogastric tube (Tyco Healthcare Group LP, Mansfield, MA) was used to administer 15 mEq sodium bicarbonate (Neogen, Lexington, KY) through the mouth into the stomach of the animal, followed by intragastric inoculation of the *S. dysenteriae* 1 strain. To confirm proper location of the tube, gas sounds in the stomach was verified by gently injecting 5 to 20 mL air while auscultating the upper abdomen using a stethoscope prior to inoculation, at which point the inoculum was injected down the tube. The orogastric tube was flushed with 10 to 20 mL of sterile saline to ensure full delivery of the bacterial inoculum.

***Clinical monitoring and treatment***

Daily monitoring for diarrhea, dysentery, fever, signs of respiratory illness, changes in food intake, or any other abnormal behavior was recorded for each macaque, including rectal temperature (Sure Temp Plus Thermometer, Welch Allyn, New York, NY) in sedated macaques (Ketamine HCl given intramuscularly at 10mg/kg) for the first 7 days and then on days 14, 28, and 56 after inoculation. Fecal samples were plated on selective media to confirm presence of *S. dysentariae* 1 strains post-immunization and post-challenge. Macaques exhibiting symptoms of *Shigella* infection (diarrhea, fever in excess of 103 °F, dysentery) were treated with antibiotics (enrofloxacin at 5 mg/kg PO or IM twice daily for 5 days, Bayer Animal Health, Shawnee Mission, KS, or ceftriaxone at 50 mg/kg IM once daily for 5 days, Apotex, Weston, FL) within 24 h of the time at which symptoms were observed. Sick macaques received intravenous fluid therapy (10 to 20 mL/kg Ringers Lactate Solution once or twice daily) if determined necessary by a facility veterinarian.

***Anti-Shigella antibody determination***

Plasma IgA and IgG antibody titers to *S. dysenteriae* 1 LPS were determined by end-point dilution ELISA. Briefly, ELISA plates (Immulon 2HB, Thermo, Milford, MA) were coated with *S. dysenteriae* 1 LPS (5 μg/ml in 0.1M Sodium Carbonate, pH 9.6) for 3 hours at 37 °C. Following coating, as well as after each subsequent incubation, plates were washed 6 times with PBS containing 0.05% Tween 20 (PBS/Tween). Plates were then blocked overnight (4 °C) using 10% dried milk in PBS. Plasma samples were evaluated in 2 fold-dilutions in 10% dried milk in PBS/Tween. IgA and IgG anti-*S. dysenteriae* 1-LPS antibodies were detected using horseradish peroxidase (HRP)-labeled goat anti-monkey Fc α-(1:2000) and γ-(1:5000) chains, respectively, (KPL, Gaithersburgh, MD) in 10% dried milk in PBS/Tween. Plasma samples, as well as secondary antibodies, were incubated for 1 hour at 37 °C. A tetramethylbenzidine (TMB) substrate solution was added (KPL, Gaithersburgh, MD) and incubated at room temperature for 15 minutes. The reaction was stopped by adding 100 μl of 1M H_3_PO_4_ and the O.D. (450 nm) was determined in an ELISA microplate reader. All samples were run in duplicate. Additionally, in each assay negative and positive (pooled sera from high-titer immunized macaques) controls were included. Linear regression curves were plotted for each for each sample and the titers were calculated as the inverse of the serum dilution that produced an O.D. of 0.2 above the blank.

***Stool collection for 16S rRNA analysis***

Stool samples were aliquoted into 0.2-0.3 gram portions and immediately frozen at -20ºC, then stored at -80ºC until further processing. Stool samples collected from the following days were used for 16S rRNA processing. Study 1: days 0, 1, 2, 7, 14, 28, 29, 30, 31, 32, 35, 56, 57, 58, 59, 60, 61, 70, and 84 (am samples). Study 2: days 0, 2, 4, 7, 10, 28, 30, and 35+ (final time point) (am samples). Study 3: days 0, 1, 2, 3, 4, 7, 14, 2and 8 (am samples). Study 4: days 0, 1, 2, 6, 7, and 14.

***Sample Preparation (Primer sequences and PCR conditions)***

The universal primer sequences were as follows, with underlined sections indicating 454 Life Sciences primers B and A, respectively: 27F, 5’-GCCTTGCCAGCCCGCTCAG-TCAGAGTTTGATCCTGGCTCAG-3’ and 338R, 5’-GCCTCCCTCG-CGCCATCAGNNNNNNNNCATTACCGCGGCTGCTGGCA-3’. PCR amplification measures are as follows for a starting volume of 50 μl: 50 ng starting DNA material, 1.0 μl of each primer (10 mM), 1.0 μl deoxyribonucleoside triphosphates (Invitrogen; 10mM), and either 0.3 μl of AmpliTaq Gold DNA polymerase with 5 μl 10x PCR buffer II and 1.5 μl MgCl2 (50mM) or AccuPrime DNA polymerase with 5 μl 10x PCR buffer II (both Invitrogen). Negative controls without DNA template were included with each PCR reaction. The following cycling parameters were used: 5 minutes (min) denaturing at 94°C followed by 30 cycles of denaturing for 30 seconds (s) at 94°C, annealing for 30 s at 55°C, and elongation for 90 s at 68°C, with a final extension period for 5 min at 72°C. Failed PCR reactions were retried at different template and PCR conditions, and if these did not work, the sample was excluded from analysis. 100 ng of the PCR product from each sample was quantified using the Quant-iT PicoGreen dsDNA assay (Invitrogen) and used for sequencing.

***Data Processing***

Genus-level classified sequence abundances were used to calculate the Shannon diversity index over time for each sample. For community type analysis, principle coordinates analysis (PCoA) was calculated using the R packages vegan and cluster, and was visualized using the R package scatterplot3d . Heatmaps were created using the R package gplots . Correlations between clinical symptoms of disease and normally rare organisms were calculated using JMP v9.0.0. Distance matrices for both short tandem repeat (STR) and MHC microsatellite data were calculated using the R package polysat . Phylip v3.69 was used to calculate phylogenetic trees from these data, and the internal R stats package was used for PCoA. The excel package GenAlEx was used to calculate MHC microsatellite allele frequencies . Network analyses for community types were created by computing the pairwise Spearman rank correlation coefficient between pivotal genera with P < 0.001 and a higher relative abundance of at least 0.1 in their corresponding community (*Streptococcus, Lactobacillus, Prevotella*, and *Enterococcus* for community types I, II, III, and IV, respectively), finding the high-order partial correlation by computing the spare inverse of the correlation matrix, and constructing a community network with the high-order partial correlation . Networks were visualized using Cytoscape 2.8.3 . Network analyses for longitudinal analysis between microbial members and immunological measurements were analyzed using the program Local Similarity Analysis (LSA) . Results were filtered by a LSA cutoff of p<0.01 and q > 0.40 and viewed in Cytoscape 2.8.3.

**Supplemental References**

1. Shipley ST, Panda A, Khan AQ, Kriel EH, Maciel M, et al. (2010) A challenge model for Shigella dysenteriae 1 in cynomolgus monkeys (Macaca fascicularis). Comp Med 60: 54-61.

2. Kanthaswamy S, Satkoski J, Georges D, Kou A, Erickson B, et al. (2008) Hybridization and stratification of nuclear genetic variation in Macaca mulatta and Macaca fascicularis. International Journal of Primatology 29: 1295-1311.

3. Wu T, Grassel C, Levine MM, Barry EM (2011) Live Attenuated Shigella dysenteriae Type 1 Vaccine Strains Overexpressing Shiga Toxin B Subunit. Infection and Immunity 79: 4912-4922.

4. Ligges U, Machler M (2003) Scatterplot3d - an R Package for Visualizing Multivariate Data. Journal of Statistical Software 8: 1-20.

5. Gregory R (2012) Gplots: various R programming tools for plotting data.

6. Clark L, Jasieniuk M (2011) POLYSAT an R package for polyploid microsatellite analysis. Molecular Ecology Resources 11: 562-566.

7. Felsenstein J (1989) PHYLIP-Phylogeny Inference Package (version 3.2). Cladistics 5: 164-166.

8. Peakall R, Smouse P (2006) GENALEX 6: genetic analysis in Excel. Population genetic software for teaching and research. Molecular Ecology Notes 6: 288-295.

9. Bien J, Tibshirani R (2011) Sparse estimation of a covariance matrix. Biometrika 98: 807-820.

10. Smoot M, Ono K, Ruscheinski J, Wang P, Ideker T (2011) Cytoscape 2.8: new features for data integration and network visualization. . Bioinformatics 27: 431-432.

11. Xia LC, Steele JA, Cram JA, Cardon ZG, Simmons SL, et al. (2011) Extended local similarity analysis (eLSA) of microbial community and other time series data with replicates. BMC Syst Biol 5 Suppl 2: S15.
